# Total hip arthroplasty versus hemiarthroplasty for independently mobile older adults with intracapsular hip fractures

**DOI:** 10.1186/s12891-019-2590-4

**Published:** 2019-05-17

**Authors:** David Metcalfe, Andrew Judge, Daniel C. Perry, Belinda Gabbe, Cheryl K. Zogg, Matthew L. Costa

**Affiliations:** 1 Nuffield Department of Orthopaedics, Rheumatology and Musculoskeletal Sciences (NDORMS), University of Oxford, Oxford, OX3 9BU UK; 2 Musculoskeletal Research Unit, Translational Health Sciences, Bristol Medical School, University of Bristol, Learning and Research Building, Level 1, Southmead Hospital, Bristol, BS10 5NB UK; 3 School of Public Health and Preventive Medicine, Monash University, Level 3, 553 St Kilda Road, Melbourne, VIC 3004 Australia; 4 Yale School of Medicine, 333 Cedar Street, New Haven, CT 06510 USA

**Keywords:** Total hip replacement, Hemiarthroplasty, Hip fractures

## Abstract

**Background:**

Displaced intracapsular hip fractures are typically treated with hemiarthroplasty (HA) or total hip arthroplasty (THA). A number of professional bodies recommend considering THA for patients that were independently mobile and cognitively intact before injury. The aim of this study was to compare the outcomes between HA and THA for independently mobile older adults with hip fractures.

**Methods:**

A systematic review and meta-analysis of RCTs was undertaken alongside analysis of a propensity score matched national cohort of older adults (aged > 60) with hip fractures. Participants were identified for the propensity score matched cohort from the National Hip Fracture Database (NHFD), which was linked to Hospital Episode Statistics (HES) and civil death registration data. The primary outcomes were 12-month dislocation, revision, and mortality. The secondary outcomes were length of stay, discharge home, unplanned re-admission, functional outcomes, and health-related quality of life.

**Results:**

Five RCTs reported higher THA dislocation but this was not statistically significant (THA risk ratio [RR] 2.77, 95% CI 0.81 to 9.48). However, THA dislocation was significantly higher in the national observational dataset (sub-distribution hazard ratio [SHR] 1.73, 95% CI 1.24 to 2.41). Meta-analysis of data from four RCTs did not identify a significant difference in terms of revision (RR 1.52, 95% CI 0.56 to 4.14). However, THA revision was significantly lower in the national dataset (SHR 0.66, 95% CI 0.48 to 0.90). Meta-analysis of data from 5 RCTs suggested higher mortality amongst patients undergoing HA (RR 0.63, 95% CI 0.38 to 1.04), which was also observed within the national registry dataset (hazard ratio 0.45, 95% CI 0.37 to 0.54).

**Conclusions:**

National clinical registries can provide important context when interpreting RCT data, which may alone be inadequate for comparing the safety profile of surgical interventions. These data suggest that THA is at significantly higher risk of dislocation but lower risk of revision within 12 months. The finding from both RCT and clinical registry data that THA is associated with lower 12-month mortality amongst the fittest patients with hip fractures requires urgent further study to determine whether or not this can be replicated in other balanced populations.

**Electronic supplementary material:**

The online version of this article (10.1186/s12891-019-2590-4) contains supplementary material, which is available to authorized users.

## Background

There are 70,000 hip fractures every year in the United Kingdom, with the total cost of care exceeding £2 billion per year. Mortality is high amongst these patients, with approximately 10% dying within 30 days of admission [1] and 30% within a year. Many survivors are unable to continue living independently and 4.5 million people worldwide are disabled every year by a hip fracture^2^.

Most intracapsular hip fractures are displaced, such that the bone fragments are no longer in continuity. Displaced intracapsular fractures are either treated with hip hemiarthroplasty (HA), where the femoral head alone is replaced, or total hip arthroplasty (THA), where the femoral head and acetabulum are both replaced. Although HA is performed more frequently, a number of organisations (such as the American Academy of Orthopaedic Surgeons [AAOS] [2] and the UK National Institute for Health and Care Excellence [NICE] [3]) recommend offering THA to selected hip fracture patients owing to perceived functional benefits. NICE recommends offering THA to patients that (1) could walk independently before the fracture (2) are not cognitively impaired and (3) are medically fit for both anaesthesia and the procedure [3]. Despite this recommendation, an international survey of orthopaedic surgeons found that 73% favour HA [4], with studies demonstrating less than a third of eligible patients actually receive THA [5]. One explanation for this discrepancy is that the evidence in support of THA is mixed. A number of small randomised controlled trials have suggested that THA is associated with better functional outcomes, fewer wound infections, and reduced need for secondary procedures [6–9]. However, THA is also a more complex procedure that requires longer surgical time, is associated with greater blood loss, and has a higher risk of subsequent dislocation [10].

It is also uncertain whether the reported benefits for THA over HA [6–9] can be replicated beyond the controlled environment of clinical trials. For example, there is a clear association between THA outcome and surgeon volume [11] and it is likely that patients will be preferentially recruited to THA trials by experienced arthroplasty surgeons. It has been suggested that increasing the number of generalist surgeons providing THA will offset the benefits of this intervention for patients with hip fractures [2]. Similarly, there are concerns that the unavailability of appropriately trained arthroplasty surgeons might delay operative treatment. Surgical delays are thought to worsen outcomes for this vulnerable patient group [12, 13] and so might even worsen outcomes for patients selected to undergo THA. It is for these reasons that the “real world” effect of increasing use of THA in the hip fracture setting has been identified as a hip fracture research recommendation by the AAOS [2].

In this study we undertook an updated meta-analysis of RCTs and used data from a comprehensive national cohort of hip fractures to provide “real world” context to the existing trial literature. Our aim was to compare the outcomes between these two procedures for independently mobile older adults with hip fractures.

## Methods

### Systematic review and meta-analysis

A scoping review identified a number of previous systematic reviews that compared HA and THA for patients with displaced intracapsular hip fractures. We therefore employed a simplified search strategy using a modification of the method first proposed by Sampson et al. [14], which has been shown to be highly sensitive (median sensitivity 100%) for identifying RCTs when applied to systematic reviews with clinically focussed research questions [15]. We used a broad search strategy: (fracture* AND (“total hip” OR hemiarthroplasty) AND “systematic review”) to search three databases (Medline 1966-, EMBASE 1947-, and CINAHL 1982-) on 1st August 2018 to identify previous systematic reviews comparing HA and THA. The reference lists of all reviews were searched and the forward citation facility in PubMed used to identify trials published after each systematic review. Trial reference lists and citations were also searched for further studies. No language restrictions were applied. The full texts of all RCTs were then screened by two authors (DM and CZ) to identify those satisfying the following inclusion criteria. A single author (DM) evaluated studies published in Chinese with help from a Chinese-speaking health economist with experience of hip fracture research. The inclusion criteria were:

- A randomised or quasi-randomised controlled trial.
- Including patients predominantly aged > 60 years with displaced intracapsular hip fractures.
- Excluding patients that had cognitive impairment or limited mobility before injury.
- Reporting dislocation, revision, mortality, unplanned re-admission, functional outcomes or health-related quality of life (using any validated scale).

Study characteristics and outcome data were extracted by one author (DM) and checked by a second (CZ). We planned to report all outcomes at 12-months for consistency. Two authors (DM and CZ) independently determined risk of bias using criteria recommended by the Cochrane Handbook [16] and resolved disagreements by consensus. These data were presented to guide judgements about the certainty of the evidence and not to determine eligibility for inclusion within meta-analyses. Data were pooled to estimate risk ratios (for categorical outcomes) and mean differences (for continuous outcomes) using the DerSimonian and Laird method for random-effects meta-analysis as high levels of between-study heterogeneity were anticipated when pooling trials from different patient populations and healthcare settings [17]. Standardised mean differences were reported when studies reported the same outcome measured on difference scales. When studies did not provide standard deviations necessary to inform confidence intervals, these were calculated from absolute *p*-values [16]. Meta-analyses were undertaken using RevMan v.5.0 (Cochrane Collaboration, Vienna, Austria). The systematic review was reported in line with the Preferred Reporting Items for Systematic Reviews and Meta-Analyses (PRISMA) statement [18] and the protocol registered prospectively in the PROSPERO database with reference CRD42018109415 [19].

### Observational “real world” data

An observational study was undertaken using a comprehensive national cohort of older adults with displaced intracapsular hip fractures to extend and contextualise the existing RCT literature. Propensity score matching was used to mimic randomisation as far as is possible using observational data.

#### Data sources

The cohort was defined using the National Hip Fracture Database (NHFD) and patient records linked to administrative data (Hospital Episode Statistics) and civil death registrations.

##### National hip Fracture Database

The National Hip Fracture Database (NHFD) is the largest hip fracture registry in the world. It is commissioned by the Healthcare Quality Improvement Partnership (HQIP) and captures data on almost all (> 95%) adults that are aged > 60 years and admitted to hospital in England, Wales, or Northern Ireland with a proximal femoral fracture [20]. There were 177 hospitals contributing data to the NHFD in 2016 [21]. Data are collected by specialist nurses in each hospital and submitted through an online platform. Submissions are linked to hospital payments through the Hip Fracture Best Practice Tariff and so completeness of core variables is high.

##### Hospital episode statistics

The Hospital Episode Statistics Admitted Patient Care (HES APC) dataset includes data on all admissions to National Health Service (NHS) hospitals or to independent sector providers that are funded by the NHS [22]. Approximately 99% of hospital activity in England is funded by the NHS [23] and so should be included within the HES APC. All activities are included that require a hospital bed (e.g. planned and emergency admissions) but outpatient and Emergency Department are excluded unless they lead to admission. The dataset includes approximately 20 million episodes of care annually from around 450 individual NHS organisations [22].

##### Office for National Statistics

The Office for National Statistics (ONS) captures data (including date and cause) on all registered deaths directly from civil registration records [24]. This dataset should therefore be complete except for the small number of cases referred to a coroner, which cannot be registered until coronial enquiries are complete and a death certificate has been issued.

#### Study population

The study period was 28th March 2011 until 4th January 2017. The start date was the earliest point at which the NHFD captured unique patient identifiers that could facilitate linkage to other datasets and the end date was chosen to facilitate 12 months follow-up. The inclusion criteria were those recommended by NICE [3]:

- All adults aged > 60.
- Displaced fracture of the femoral neck that was deemed unsuitable for internal fixation.
- Independently mobile or using a single stick before injury.
- Medically fit to undergo hip arthroplasty, defined as an American Society of Anaesthesiologists [ASA] grade < 2 [5].
- Patients without substantial cognitive impairment, defined as an Abbreviated Mental Test Score (AMTS) > 8 [5].

We excluded patients that presented to hospitals in Wales, Northern Ireland, and the Isle of Man as HES APC only captures data from hospitals in England. Cases were also excluded if they could not be positively matched to records within HES APC based on their NHS number, sex, date of birth, and full post-code.

#### Outcomes

The primary outcomes were dislocation, revision, and mortality within 12-months. The secondary outcomes were surgical delay, length of stay, discharge to own home, and re-admission within 30 days. Surgical delay, length of stay, and discharge destination were available directly from the NHFD. Revision operations were identified from HES APC and defined by OPCS v4 (OPCS4) procedure codes previously used in other studies and incorporating codes recommended for this purpose by the UK National Joint Registry [25] (Additional file 1). Dislocation OPCS4 codes were identified by manual searches using disloc*, manipula*, and reduc* (Additional file 1).

#### Statistical analysis

##### Matching

We calculated propensity scores that represented the estimated probability of each patient undergoing THA based on characteristics that are known to be associated with outcome in this population: age, sex, pre-injury mobility status, admission source, American Society of Anaesthesiologists (ASA) physical status grade, Charlson Co-morbidity Index (Additional file 1), Abbreviated Mental Test Score (AMTS), and Index of Multiple Deprivation (IMD) [26]. The model was otherwise specified iteratively to achieve the best possible match, as judged by visual inspection of the distribution of propensity scores after matching and plots of co-variables against propensity scores by treatment status. We also undertook post-estimation statistical checks [27], which included t-tests for differences in means and confirmation that the standardised mean difference for each co-variable between the groups was < 1% [28]. The final model utilised 1:1 nearest neighbour matching with a 0.02 calliper (as recommended by Austin [29]), no replacement, and the common support restriction. All subsequent descriptive, regression, and survival analyses were confined to the propensity score matched groups.

##### Descriptive statistics

Categorical variables were compared using Chi-square tests and non-normally distributed continuous variables using the Kruskall-Wallis one-way analysis of variance test. Length of stay data were only analysed for the proportion of patients that were discharged alive from hospital to prevent left skew caused by early deaths.

##### Survival analysis

Kaplan-Meier estimates were plotted with 95% confidence intervals for cumulative survival free from unplanned secondary procedures. The proportional hazards assumption was tested by visual examination and statistical assessment of the relationship between event time and Schoenfeld residuals. The proportional hazards assumption was satisfied and so we used Cox regression models fitted with our primary outcome (dislocation and/or revision) as the independent variable. Mortality is high in this population and so we undertook a sensitivity analysis using competing risks regression models with death specified as the competing risk. Competing risks regression models were also fitted for dislocation and revision as individual events. The co-variables for all regression models were those described above as the basis for propensity score matching, which include five of the six used routinely in the NHFD for case mix adjustment [30]. The sixth NHFD case mix co-variable (i.e. fracture type) was not used because only patients with displaced intracapsular hip fractures were included in this study. Year of fracture was included as an ordinal variable within regression models to account for the possibility of changing outcomes over time.

##### Multivariable regression

Multivariable logistic regression was used to adjust for residual imbalance between the two groups in respect of discharge to own home and 30-day re-admission. The co-variables were as specified above. Length of stay data conformed to a gamma distribution and so were adjusted using generalized linear models (GLM) together with post-estimation calculations of average marginal effects to yield predicted mean differences and 95% confidence intervals. Logistic regression and GLMs utilised cluster-robust standard errors and robust variance estimators [31] to account for the lack of independence between matched records [32].

Propensity score matching was achieved using the MatchIt application for R (R Foundation for Statistical Computing, Vienna, Austria). All subsequent analyses were undertaken using StataIC v.15 (StataCorp, College Station, TX, USA). Two tailed *p* < 0.05 was adopted a priori as the threshold for statistical significance.

## Results

### Meta-analysis of randomised trials

There were 11 previous systematic reviews but none reported analyses limited to patients that were cognitively intact and independently mobile before injury (Additional file 2: Figure S1). The 11 earlier reviews included 16 trial reports, which presented data from 14 individual RCTs. Eight RCTs did not satisfy the restricted inclusion criteria of this systematic review, e.g. they did not exclude patients with cognitive impairment or limited mobility. One study could not be retrieved despite extensive attempts. The reasons for excluding each RCT are shown in Additional file 2: Table S1. Five randomised controlled trials satisfied the eligibility criteria for this review (Additional file 2: Table S2). Two were based in the UK [33, 34] and one each in Sweden [35], Italy [36], and the USA [9]. A further eligible RCT is on-going [37]. Characteristics of the RCTs and risk of bias assessments are described in Additional file 2. All the RCTs used adequate random sequence generation techniques and were judged to be at low risk of attrition bias as loss to follow-up was low. However, no RCT sought to blind patients, personnel, or outcome assessors.

### Observational “real world” data

**Fig. 1:**
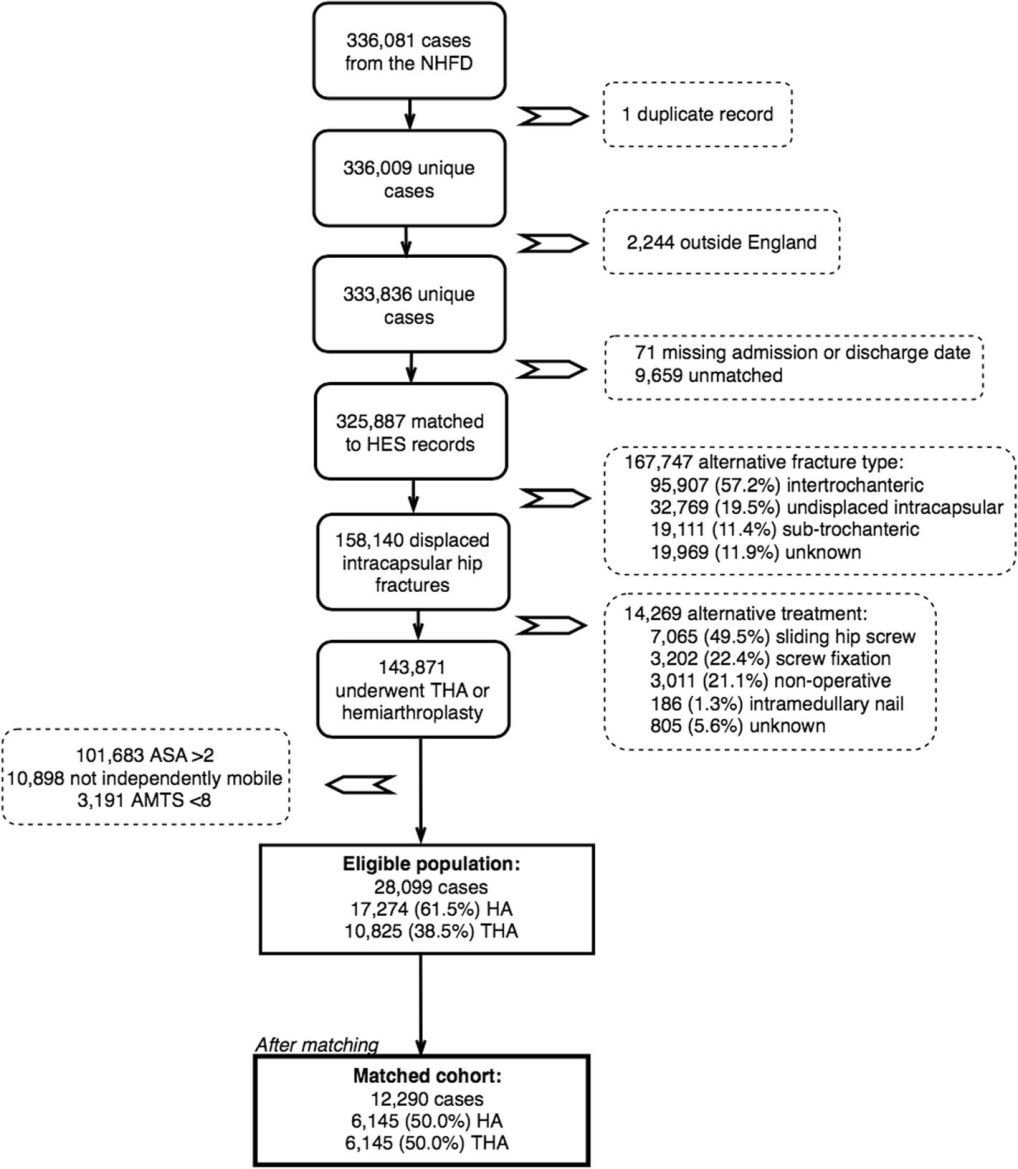
A flow diagram showing inclusion of cases within the study

There were 143,871 patients with displaced intracapsular hip fractures that underwent HA or THA and could be matched to a record within HES APC (Fig. 1). 28,099 (19.5%) satisfied the pre-specified inclusion criteria, i.e. ASA < 2, AMTS > 8, and independently mobile. The groups initially varied considerably in terms of baseline characteristics (Additional file 3). After propensity score matching, 12,290 cases were selected for further analysis. Table 1 shows that the baseline characteristics of the matched groups were similar. The distribution of propensity scores was also improved after matching (Additional file 3).

**Table 1:** Characteristics of the matched population

	Hemiarthroplasty	Total hip arthroplasty	Total	P
Age^c^	77 (72–81)	77 (73–81)	77 (73–81)	0.571^a^
Sex^d^				
Male	1347 (21.9%)	1321 (21.5%)	2668 (21.7%)	
Female	4798 (78.1%)	4824 (78.5%)	9622 (78.3%)	0.569^b^
ASA^c^	2 (2–2)	2 (2–2)	2 (2–2)	0.675^a^
Pre-injury mobility^d^				
Independently mobile	5308 (86.7%)	5326 (86.7%)	10,634 (86.7%)	
Mobile indoors with one aid	837 (13.6%)	819 (13.3%)	1656 (13.5%)	0.634^b^
AMTS^c^	10 (10–10)	10 (10–10)	10 (10–10)	0.457^a^
Admission source^d^				
Own home	6071 (98.8%)	6092 (99.1%)	12,163 (99.0%)	
Rehabilitation unit	8 (0.1%)	2 (0.0%)	10 (0.1%)	
Residential/nursing home	37 (0.6%)	18 (0.3%)	55 (0.5%)	
Acute hospital	29 (0.5%)	33 (0.5%)	62 (0.5%)	0.015^b^

*Median (interquartile range); **number (percentage); ^a^Kruskall-Wallis one-way analysis of variance; ^b^Chi2 test

### Primary outcomes

#### Dislocation

**Fig. 2:**
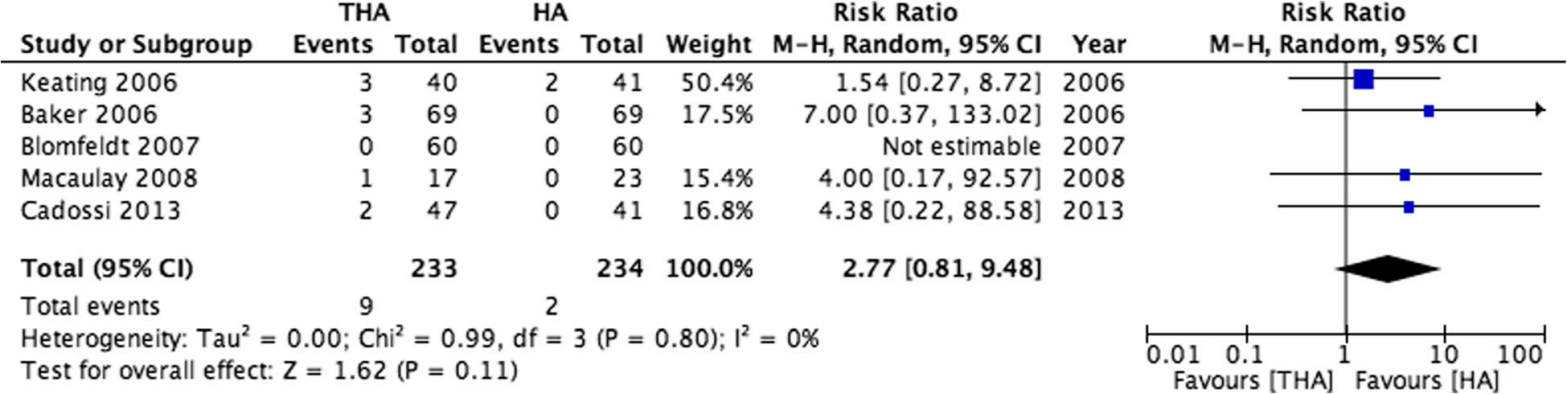
A forest plot showing risk of 12-month dislocation within eligible clinical trials

All five RCTs reported risk of dislocation. Although the pooled effect estimate suggested higher risk of dislocation amongst those undergoing THA, this was not significant (THA 9/233 [3.9%] versus HA 2/234 [0.9%], RR 2.77 [95% 0.81 to 9.48], Fig. 2). Within the propensity score matched cohort, those undergoing THA were significantly more likely to dislocate than those with HA (1.6% versus 0.9%, X^2^ *p* < 0.001). This finding persisted when adjusting for co-variables in a competing risks regression model (THA sub-distribution hazard ratio [SHR] 1.73, 95% CI 1.24 to 2.41, see Table 2).

**Table 2:** Clinical outcomes for patients by operation

	Hemiarthroplasty	Total hip arthroplasty	P
Primary outcomes			
Dislocation (12 months)	57 (0.9%)	96 (1.6%)	0.002^a^
	THA sub-distribution hazard ratio 1.73 (CI 1.24 to 2.41)^b^		
Revision (12 months)	106 (1.7%)	67 (1.1%)	< 0.001^a^
	THA sub-distribution hazard ratio 0.66 (0.48 to 0.90)^b^		
Mortality (12 months)	58 (5.5%)	159 (2.6%)	< 0.001^a^
	THA hazard ratio 0.45 (95% CI 0.37 to 0.54)^c^		
Secondary outcomes			
Surgical delay (hours)^d^	22.2 (17.8–29.0)	23.9 (18.9–40.6)	< 0.001^e^
Length of stay (days)^d^	10 (7–15)	9 (7–13)	< 0.001^e^
	THA predicted mean difference − 1.92 (95% CI −2.30 to −1.55) days^f^		
Discharge home	5017 (80.7%)	5519 (88.6%)	< 0.001^a^
	THA adjusted odds ratio 1.77 (95% CI 1.58 to 1.99)^g^		
Re-admission (30-days)	361 (5.9%)	356 (5.8%)	0.847^a^
	THA adjusted odds ratio 0.96 (95% CI 0.82 to 1.11)^g^		

^a^Chi^2^ test; ^b^Competing risks regression model; ^c^Royston-Parmar flexible parametric model; ^d^Median (interquartile range); ^e^Kruskall-Wallis one-way analysis of variance; ^f^Predicted mean difference from a generalized linear model; ^g^Multivariable logistic regression model

#### Revision

**Fig. 3:**
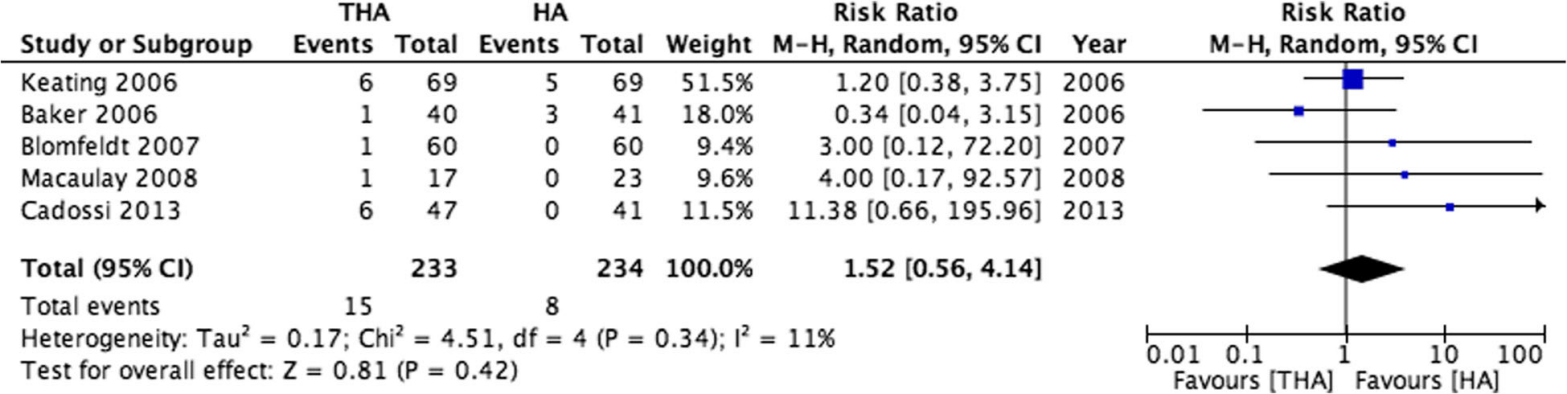
A forest plot showing risk of 12-month revision within eligible clinical trials

All five RCTs reported risk of revision [9, 33–36]. The pooled effect estimate was initially in favour of HA, although this association was not statistically significant (HA 8/234 [3.4%] versus 15/233 [6.4%], RR 1.52 [95% CI 0.56 to 4.14], Fig. 3). The association also diminished when the data reported by Cadossi et al. [36] were excluded as these authors had trialled a non-standard THA prosthesis and reported an unusually high revision rate (HA 8/193 [4.1%] versus 9/186 [4.8%], RR 1.16 [95% CI 0.46 to 2.91]). However, within the propensity score matched cohort*,* a greater proportion of *HA* patients underwent revision surgery within the subsequent 12 months than THA (1.7% versus 1.1%, X^2^ *p* < 0.001). This finding persisted when adjusting for co-variables in a competing risks regression model (THA SHR 0.66, 95% CI 0.48 to 0.90).

#### Mortality

**Fig. 4:**
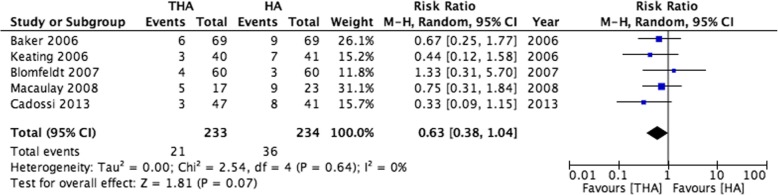
A forest plot showing risk of 12-month mortality within eligible clinical trials

**Fig. 5:**
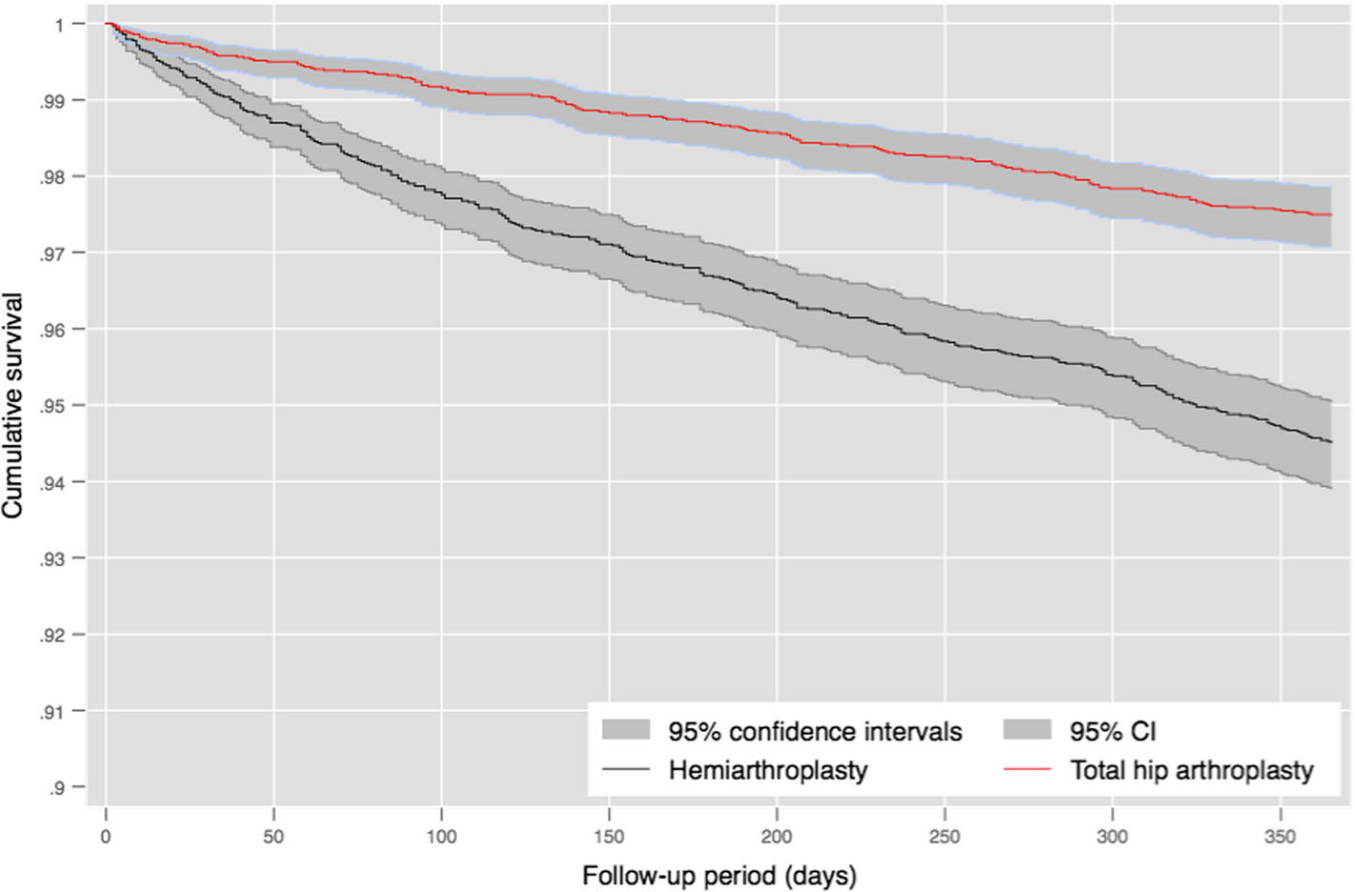
Kaplan-Meier plot showing mortality for patients in the propensity score matched cohort

Four RCTs reported mortality at 12 months and one at 6 months. A higher proportion of patients undergoing HA died (36/234, 15.4%) than those in the THA group (21/233, 9.0%, RR 0.63, 95% CI 0.38 to 1.04, Fig. 4). Within the propensity score matched cohort, 12-month mortality was higher in the HA group (5.4% versus 2.6%, X^2^ *p* < 0.001) and this persisted within a multi-level flexible parametric survival model (hazard ratio 0.45, 95% CI 0.37 to 0.54). Twelve-month mortality within the observational cohort is illustrated by a Kaplan-Meier plot in Fig. 5.

### Secondary outcomes

#### Time to surgery

Two RCTs [33, 36] (164 patients) reported no difference in time to surgery between THA and HA (THA mean difference − 0.44 [95% CI − 0.93 to 0.05]). Within the propensity score matched cohort, patients underwent HA more promptly than THA (median 22.2 [interquartile range (IQR) 17.8–29.0] hours versus 23.9, 18.9–40.6 h, Kruskall-Wallis *p* < 0.001).

#### Duration of surgery

All five RCTs (462 patients) reported surgical duration. Although THA took longer than HA, and this difference was statistically significant, the absolute effect was small (mean difference 15.0 [95% CI 6.4 to 23.7] minutes). Duration of surgery was not available from the propensity score matched cohort.

#### Length of stay

Two RCTs (123 patients) reported length of stay and there was no intervention effect on this outcome (THA mean difference 1.50 [95% CI 0.00 to 3.00] days). In the propensity score matched cohort, patients undergoing HA stayed in hospital longer than those undergoing THA (median 10 [IQR 7–15] versus 9 [7–13] days, Kruskall-Wallis, *p* < 0.001). When adjusting for co-variables within a generalised linear model, patients undergoing THA experienced a shorter length of stay (predicted mean difference − 1.92 [95% CI − 2.30 to − 1.55] days).

#### Discharge destination

No RCT reported discharge destination as an outcome. Within the propensity score matched cohort, a smaller proportion of patients undergoing HA were discharged to their own home than THA (80.7% versus 88.6%, X^2^ p < 0.001). Within a multivariable logistic regression model, those undergoing THA also had higher odds of being discharged to their own home (adjusted odds ratio [aOR] 1.77, 95% CI 1.58 to 1.99).

#### 30-day readmission

No RCT reported unplanned readmission to hospital as an outcome. Within the propensity score matched cohort, there was no statistically significant difference in 30-day re-admission between the two groups (HA 5.9% versus THA 5.8%, X^2^ *p* = 0.847), and this finding persisted within a multivariable logistic regression model (aOR 0.96, 95% CI 0.82 to 1.11).

#### Hip functional outcomes

All five RCTs reported joint-specific functional outcomes measured at 12-months. Three studies used the Harris Hip Score [9, 35, 36] (234 patients) and one each used the Oxford Hip Score [33] (81 patients), Western Ontario and McMaster Universities Osteoarthritis Index (WOMAC) [9] (40 patients), and a bespoke hip questionnaire [34] (138 patients). Higher scores on all of these measures reflect better outcomes except for the Oxford Hip Score in which a higher score represents *worse* function. There were no differences in terms of total score (THA standardised mean difference [SMD] 0.17 [95% CI − 0.20 to 0.53]) or either the pain (− 0.01 [− 0.49 to 0.48]) or function (0.18 [− 0.03 to 0.39]) domains. The only study using the Oxford Hip Score reported a difference between the groups in favour of THA (THA mean difference − 3.50 [95% CI − 6.66 to − 0.34]). However, the only study reporting data from a “Timed Up and Go” (TUG) test [9] (40 patients) – which measures the time that it takes a patient to rise from a chair, walk three metres, turn around, walk back to the chair, and sit down – did not find a difference between the groups (THA mean difference − 0.70 [95% CI − 8.01 to 6.61] seconds).

#### Health-related quality of life

Two studies [9, 33] (121 patients) reported components of the Short Form (36) Health Survey (SF-36) and one the EQ-5D [34] (183 patients). There were no differences in the mental (THA mean difference 2.30 [95% CI − 8.57 to 13.18]) or physical (2.98 [− 0.89 to 6.85]) component summary scores of the SF-36 or the EQ-5D (0.10 [0.00 to 0.20]) utility score.

## Discussion

No previous meta-analysis has reported data limited to the fittest patients with hip fractures, which are the patients that national guidelines recommend should be considered for THA [2, 3]. This study identified five RCTs that compared HA and THA amongst independently mobile older adults with displaced intracapsular hip fractures [9, 33–36]. These trials were typically small (median 89 patients) single-centre studies that were limited by few events (pooled totals 11/467 [2.4%] dislocations, 23/467 [4.9%] revisions, and 57/467 [12.2%] deaths). No individual trial reported differences in outcomes and it is even possible that the pooled analyses were underpowered to detect important differences between the groups. We therefore analysed data from the largest available cohort of hip fracture patients and used propensity score matching to replicate randomisation as far as is possible using observational data. The observational data confirmed the non-significant trend reported by RCTs that THA has a higher risk of 12-month dislocation. However, we found a 33% lower risk of 12-month revision for THA patients, which is contrary to the RCT finding of “no difference” between the groups observed in the RCTs.

Importantly, we identified a 58% lower risk of 12-month mortality for patients undergoing THA. Although this may reflect residual confounding, a similar association was evident from the meta-analysis of data from all five trials. One possibility is that the increased power available from the observational cohort has confirmed an association initially evident in the RCT data. This finding would however need to be replicated in further studies before it could be used to guide surgical decisions.

We also presented data that has not previously been reported by RCTs, including time to surgery, length of stay, discharge destination, and 30-day re-admission. Our study found that patients undergoing THA waited longer for an operation (approximately 1.7 h), although this delay is unlikely to be clinically significant. Although the AAOS have expressed concern that increased provision of THA might lead to operative delays [2], our study suggests that hospitals in England are providing THA within a timeframe that is comparable to HA. We found that THA was associated with a shorter length of stay (by approximately 1.9 days) and increased odds of discharge home. However, there was no difference between the groups in terms of 30-day re-admission.

There was mixed evidence from the RCTs as to whether or not functional outcomes or health-related quality of life vary between the groups at 12-months. The meta-analyses did not identify any statistically significant differences, although one study reported significantly better Oxford Hip Scores in the THA group [33]. There is however evidence to suggest that the functional benefits of THA become more pronounced over a number of years follow-up [7].

There is one on-going RCT [37] that might – either in isolation or when combined with data from previous trials – report sufficient events to identify differences between the two operations. However, the AAOS has expressed concern that the benefits of THA might not be generalisable beyond the controlled environment of clinical trials [2]. The RCTs identified in this study were all based in large academic centres and two [35, 36] specified that operations were only performed by experienced arthroplasty surgeons. Observational datasets can provide important context for RCT findings as they reflect “real world” practice in which operations may also be performed in smaller orthopaedic units, by generalist orthopaedic surgeons, and by trainees. It is therefore reassuring that, although the propensity score matched cohort mirrored the RCT participants in terms of HA dislocation rate (both 0.9%), the THA dislocation rate was *lower* in the observational cohort than reported by trials (1.6% versus 3.9%). There were also fewer revisions identified in the propensity score matched cohort than were reported by the RCTs (THA 1.1% versus 1.7%; RCT 4.8% versus 4.1%). Although it is possible that some dislocations and revision procedures were not captured by the linked dataset, our findings are similar to those of a recent population-based study from Canada [11]. These authors reported findings that were the same in both magnitude and direction (THA dislocation 1.9% versus 0.8%; revision 0.4% versus 2.3%) as observed in our study. It is therefore possible that contemporary prostheses perform better (in terms of major hip complications) than those used in trials undertaken between 2006 and 2013. Our findings do not support the hypothesis that THAs undertaken outside RCTs are more prone to dislocation and early revision.

### Limitations

There are a number of limitations to our approach. First, although extensive attempts were made to account for case-mix differences within the cohort study, it is possible that some findings were subject to residual confounding, which would be expected to bias findings against HA as surgeons are encouraged to reserve THA for the fittest patients. However, it is important that a similar signal was observed within the RCT data, which should be much more resistant to confounding. Second, as the NHFD was established to audit hip fracture care, it does not collect some variables (e.g. surgical approach) that might be found in a dedicated hip fracture registry. Surgical approach is known to be associated with dislocation [38] and this may be a further source of confounding. Third, coding errors are inevitable within the NHFD and HES. However, the NHFD has almost complete case capture and all re-admissions to hospitals in England over the subsequent 12 months should have been represented within HES. It is nevertheless possible that some events will not have recorded within HES. Although all arthroplasty revision procedures would have been within the context of an inpatient admission, some dislocations (e.g. those reduced and discharged home directly from the Emergency Department) might not have been captured by our study. Previous work in other surgical settings has found that OPCS4 codes in HES can reliably be used to identify some operations, although this can vary substantially between procedures [39]. However, a range of codes were used to define “revision surgery” and this selection might have influenced the findings. Nevertheless, our dislocation and revision rates were reassuringly similar to those reported by a recent population-based study from Canada [11]. Finally, there is evidence that the functional and health-related quality of life benefits of THA only become apparent after a number of years [7]. This study sought to compare early complications and chose 12-month follow-up as a means of directly comparing RCT findings with those from a national cohort of comparable patients with hip fractures. It is however possible that our meta-analyses understated functional benefits of THA in this population.

## Conclusion

This study found that concerns about increased provision of THA leading to clinically significant delays for older adults with hip fractures are unfounded. Similarly, there was not any evidence that dislocation or revision rates are higher in England outside the context of clinical trials. The finding of increased mortality amongst patients undergoing HA requires urgent further study to determine whether or not this can be replicated in other balanced populations.

## Additional files

Additional file 1: Codes for defining Charlson co-morbidities* (DOCX 19 kb)

Additional file 2: **Figure S1.** PRISMA flow diagram showing identification of randomised and quasi-randomised controlled trials from previous systematic reviews. **Table S1.** Characteristics of excluded studies. **Table S2.** Characteristics of included studies. **Table S3.** Risk of bias assessments for included studies. (DOCX 321 kb)

Additional file 3: **Table S1.** Characteristics of the unmatched population. **Figure S1.** Histograms showing the distribution of propensity scores before and after matching. **Figure S2.** Quantile-quantile plots of co-variables between the two groups before and after matching. *Data from populations with the same empirical distribution will lie along the 45 degree reference line.* **Figure S3.** Co-variables plotted against propensity scores by treatment status. *If the two are identical, this indicates that the groups have the same mean for each value of the propensity score and so are well matched.* **Figure S4.** A jitter plot showing the overall distribution of propensity scores for both matched and unmatched records. (DOCX 689 kb)

## Abbreviations

AAOS: American Academy of Orthopaedic Surgeons
AMTS: Abbreviated mental test score
aOR: Adjusted odds ratio
ASA: American Society of Anesthesiologists
CAG: Confidentiality advisory group
CCI: Charlson co-morbidity index
CI: Confidence interval
DARG: Data access request group
GAFReC: Governance arrangements for Research Ethics Committees
GLM: Generalised linear model
HA: Hemiarthroplasty
HES: APC Hospital Episode Statistics Admitted Patient Care
HES: Hospital Episode Statistics
HQIP: Healthcare quality improvement partnership
HR: Hazard ratio
IMD: Index of multiple deprivation
IQR: Interquartile range
NHFD: National hip fracture database
NHS: National Health Service
ONS: Office for National Statistics
OPCS: Office of Population Censuses and Surveys
PRISMA: Preferred reporting items for systematic reviews and meta-analyses
RCT: Randomised controlled trial
RR: Risk ratio
SHR: Standardized sub-distribution hazard ratio
SMD: Standardised mean difference
THA: Total hip arthroplasty
TUG: Timed up and go
WOMAC: Western Ontario and McMaster Universities Osteoarthritis Index
