# Supplementary material for: Total hip arthroplasty versus hemiarthroplasty for independently mobile older adults with intracapsular hip fractures
Source: BMC Musculoskelet Disord. 2019 May 17;20:226. doi: 10.1186/s12891-019-2590-4 (PMC6525472; doi:10.1186/s12891-019-2590-4)
Supplement: Supplementary file 1 — Codes for defining Charlson co-morbidities* (DOCX 19 kb) [file 12891_2019_2590_MOESM1_ESM.docx]

**Additional file 1**

**Codes for defining Charlson co-morbidities***

| **Charlson diagnosis categories based on ICD-10-CM diagnoses** | |
| --- | --- |
| **Category** | **ICD-10 codes** |
| ***Score = 1***  Myocardial infarction  Congestive heart failure  Peripheral vascular disease  Dementia  Cerebrovascular disease  Chronic lung disease  Connective tissue disease  Peptic ulcer  Chronic liver disease  Diabetes | I21, I22  I50.0  I70-I73  F00-F03  I60-67  J41-47  M05-06; M08; M15-19; M35-36  K25-28  K70.0, K76.0, K76.1  E10-E14, 4th digit X,0,1,9 |
| ***Score = 2***  Hemiplegia  Moderate or severe kidney disease  Diabetes with end organ damage  Tumour  Leukaemia  Lymphoma | G81  N17-19  E10-E14, 4th digit 2-8; N083  C00-C76; C80; C88; C90.0, C90.2; C96; C97; D00-49  C90.1; C91-95  C81-85 |
| ***Score = 3***  Moderate or severe liver disease | K70-76 without K70.0, K76.0, K76.1 |
| ***Score = 6***  Metastatic cancer  AIDS | C77-79  B20-B23 |

*Based on Charlson ME, Pompei P, Ales KL, MacKenzie CR. A new method of classifying prognostic comorbidity in longitudinal studies: development and validation. J Chronic Dis. 1987;40(5):737-83.

**Codes for Dislocation**

**Dislocation = (Group 1 OR Group 2) OR (Group 3 AND Group 4)**

| **Group 1** | |
| --- | --- |
| **ICD10 code** | **Description** |
| T84.020  T84.020A  T84.020D  T84.020S  T84.021  T84.021A  T84.021D  T84.021S | Dislocation of internal right hip prosthesis  Dislocation of internal right hip prosthesis – initial encounter  Dislocation of internal right hip prosthesis – subsequent encounter  Dislocation of internal right hip prosthesis – sequela  Dislocation of internal left hip prosthesis  Dislocation of internal left hip prosthesis – initial encounter  Dislocation of internal left hip prosthesis – subsequent encounter  Dislocation of internal left hip prosthesis - sequela |

| **Group 2** | |
| --- | --- |
| **OPCS4 code** | **Description** |
| W396  W485 | Closed reduction of dislocated total prosthetic replacement of hip joint  Closed reduction of dislocated prosthetic replacement of head of femur |

| **Group 3** | |
| --- | --- |
| **OPCS4 code** | **Description** |
| W65  W658  W659  W66  W662  W668  W669  W67  W676  W678  W679 | Primary open reduction of traumatic dislocation of joint  Other specified primary open reduction of traumatic dislocation of joint  Unspecified primary open reduction of traumatic dislocation of joint  Primary closed reduction of traumatic dislocation of joint  Primary closed reduction of traumatic dislocation of joint and skeletal traction NEC  Other specified primary closed reduction of traumatic dislocation of joint  Unspecified primary closed reduction of traumatic dislocation of joint  Secondary reduction of traumatic dislocation of joint  Remanipulation of traumatic dislocation of joint  Other specified secondary reduction of traumatic dislocation of joint  Unspecified secondary reduction of traumatic dislocation of joint |

| **Group 4** | |
| --- | --- |
| **OPCS4 code** | **Description** |
| Z843  Z761  Z756 | Hip joint  Head of femur  Acetabulum |

**Codes for revision***

**Revision = Group 1 OR (Group 2 AND Group 5) OR (Group 3 AND Group 6) OR (Group 4 AND Group 5 AND Group 6).**

| **Group 1** | | |
| --- | --- | --- |
| **OPCS4 code** | **Description** |  |
| W370  W372  W373  W374  W380  W382  W383  W384  W392  W393  W395  W462  W472  W482  W932  W933  W940  W942  W943  W952  W953  W954 | Total prosthetic replacement of hip joint using cement, Conversion from previous cemented total prosthetic replacement of hip joint  Conversion to total prosthetic replacement of hip joint using cement  Revision of total prosthetic replacement of hip joint using cement  Revision of one component of total prosthetic replacement of hip joint using cement  Total prosthetic replacement of hip joint not using cement, Conversion from previous uncemented total prosthetic replacement of hip joint  Conversion to total prosthetic replacement of hip joint not using cement  Revision of total prosthetic replacement of hip joint not using cement  Revision of one component of total prosthetic replacement of hip joint not using cement  Conversion to total prosthetic replacement of hip joint NEC  Revision of total prosthetic replacement of hip joint NEC  Revision of one component of total prosthetic replacement of hip joint NEC  Conversion to prosthetic replacement of head of femur using cement  Conversion to prosthetic replacement of head of femur not using cement  Conversion to prosthetic replacement of head of femur NEC  Conversion to hybrid prosthetic replacement of hip joint using cemented acetabular component  Revision of hybrid prosthetic replacement of hip joint using cemented acetabular component  Conversion from previous hybrid prosthetic replacement of hip joint using cemented femoral component  Conversion to hybrid prosthetic replacement of hip joint using cemented femoral component  Revision of hybrid prosthetic replacement of hip joint using cemented femoral component  Conversion to hybrid prosthetic replacement of hip joint using cement NEC  Revision of hybrid prosthetic replacement of hip joint using cement NEC  Attention to hybrid prosthetic replacement of hip joint using cement NEC |  |

| **Group 2** | |
| --- | --- |
| **OPCS4 code** | **Description** |
| W522  W523  W532  W533  W542  W543  W572  W574  W582 | Conversion to prosthetic replacement of articulation of bone using cement NEC  Revision of prosthetic replacement of articulation of bone using cement NEC  Conversion to prosthetic replacement of articulation of bone not using cement NEC  Revision of prosthetic replacement of articulation of bone not using cement NEC  Conversion to prosthetic replacement of articulation of bone NEC  Revision of prosthetic replacement of articulation of bone NEC  Primary excision arthroplasty of joint NEC  Conversion to excision arthroplasty of joint  Revision of resurfacing arthroplasty of joint |

| **Group 3** | |
| --- | --- |
| **OPCS4 code** | **Description** |
| W394 | Attention to total prosthetic replacement of hip joint NEC |

| **Group 4** | |
| --- | --- |
| **OPCS4 code** | **Description** |
| W544 | Attention to prosthetic replacement of articulation of bone NEC |

| **Group 5** | |
| --- | --- |
| **OPCS4 code** | **Description** |
| Z843  Z761  Z756 | Hip joint  Head of femur  Acetabulum |

| **Group 6** | |
| --- | --- |
| **OPCS4 code** | **Description** |
| Y032  Y037 | Renewal of prosthesis in organ NOC  Removal of prosthesis from organ NOC |

*OPCS4 codes identified from the National Joint Registry (NJR) Centre, January 2012. http://www.njrcentre.org.uk/njrcentre/Portals/0/Documents/OPCS4%20Procedure%20Codes%20used%20in%20NJR%20Annual%20Report.pdf?ver=2012-02-15-165150-000.
