# Supplementary material for: Total hip arthroplasty versus hemiarthroplasty for independently mobile older adults with intracapsular hip fractures
Source: BMC Musculoskelet Disord. 2019 May 17;20:226. doi: 10.1186/s12891-019-2590-4 (PMC6525472; doi:10.1186/s12891-019-2590-4)
Supplement: Supplementary file 2 — Figure S1. PRISMA flow diagram showing identification of randomised and quasi-randomised controlled trials from previous systematic reviews. Table S1. Characteristics of excluded studies. Table S2. Characteristics of included studies. Table S3. Risk of bias assessments for included studies. (DOCX 321 kb) [file 12891_2019_2590_MOESM2_ESM.docx]

Additional file 2

**Figure S1:** PRISMA flow diagram showing identification of randomised and quasi-randomised controlled trials from previous systematic reviews.


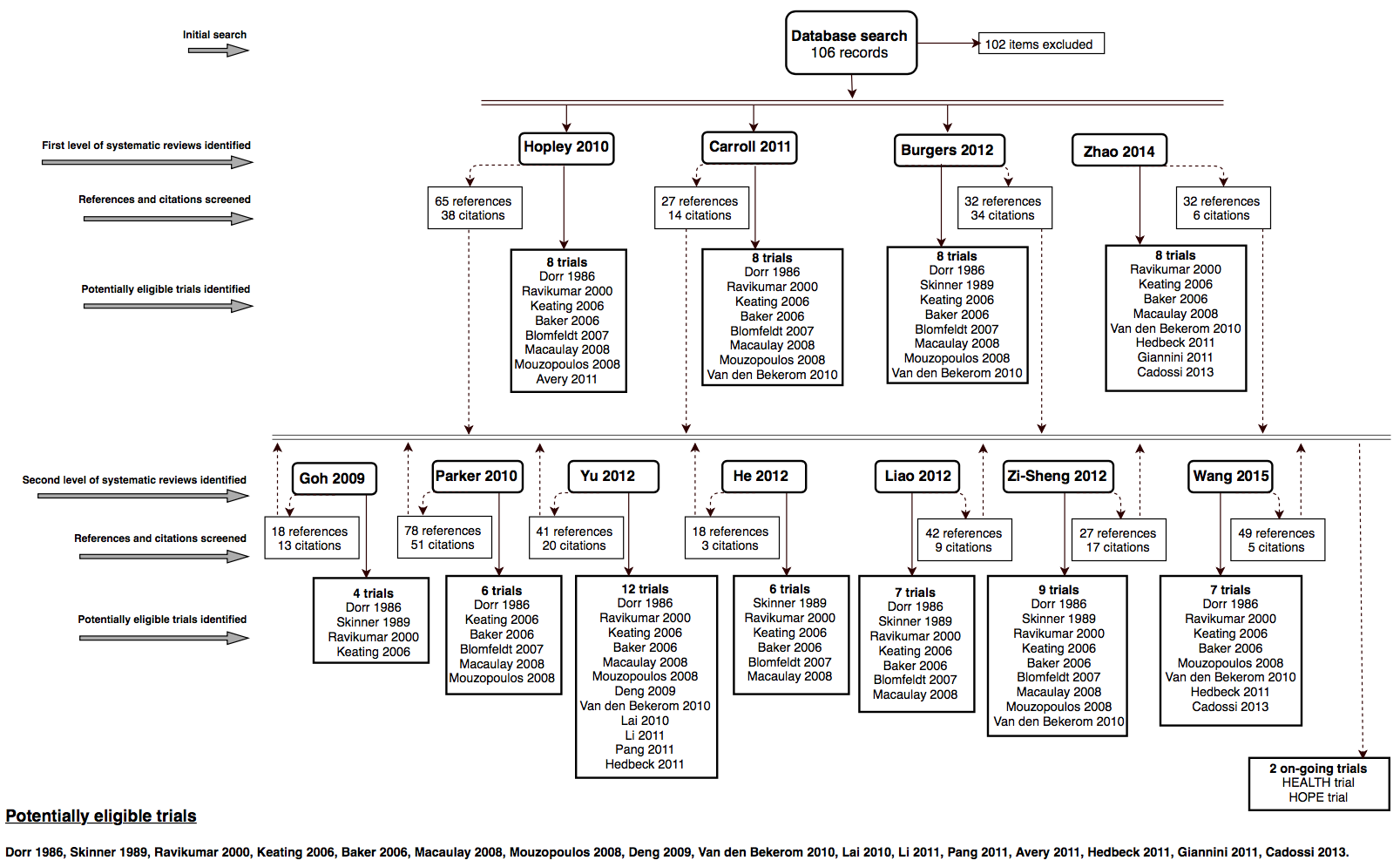


| **Table S1: Characteristics of excluded studies** | |
| --- | --- |
| **Study** | **Reason for exclusion** |
| Dorr* | Included all patients with displaced subcapital fractures – no clear exclusion criteria. |
| Skinner* | Included all patients with displaced subcapital fractures – no exclusions based on cognitive or mobility status. |
| Ravikumar* | Included all patients with displaced subcapital fractures – no exclusions based on cognitive or mobility status. |
| Mouzopoulos* | Included all patients with displaced subcapital fractures – no exclusions based on cognitive or mobility status. |
| Deng | Included all patients with displaced intracapsular fractures – no exclusions based on cognitive or mobility status. |
| Van den Bekerom | Included patients aged >70 and only excluded those that could not consent to participation and/or were bedbound. |
| Lai | Included all patients with displaced intracapsular fractures – no exclusions based on cognitive or mobility status. |
| Li | Included all patients with displaced intracapsular fractures – no exclusions based on cognitive or mobility status. |
| Pang | Could not be obtained**. |

*Quasi-randomised controlled trials; **Unable to obtain study despite attempts to contact authors, assistance from the China Centre Library at the University of Oxford, requests to The British Library, and attempts to source these articles from collaborating libraries in both the UK and the USA.

| **Table S2: Characteristics of included studies** | | | | | | | | |
| --- | --- | --- | --- | --- | --- | --- | --- | --- |
| **Study** | **Setting** | | **Participants** | | **Intervention** | | | |
|  | **Country** | **Centres** | **Total** | **Inclusion criteria*** | **Hemiarthroplasty** | | **Total hip arthroplasty** | |
|  |  |  |  |  | **Total** | **Type** | **Total** | **Type** |
| Baker 2006  (+ Avery) | UK | 1 | 81 | Age >60; normal AMTS; ability to walk >0.8km independently. | 41 | Direct lateral approach. Cemented femoral component with an Endo Femoral Head (Zimmer). | 40 | Direct lateral approach. Cemented femoral component with an all-polyethylene cemented acetabular cup (Zimmer). |
| Keating 2006 | UK | 11 | 138 | Normal cognitive function (AMTS >6); independently mobile; recruiting surgeon believes both interventions acceptable. | 69 | Approach and prosthesis at discretion of the operating surgeon. | 69 | Approach and prosthesis at discretion of the operating surgeon. |
| Blomfeldt 2007  (+ Hedbeck 2011) | Sweden | 1 | 120 | Age 70-90 years; absence of severe cognitive dysfunction assessed using the Short Portable Mental Status Questionnaire; independent walking ability. | 60 | Modified Hardinge approach. Cemented Exeter femoral component. | 60 | Modified Hardinge approach. Cemented Exeter femoral component with a bipolar head or an OGEE cemented acetabular component. |
| Macaulay 2008 | USA | 5 | 40 | Age >50; independently mobile; score >23/30 on the Folstein Mini Mental State Examination. | 23 | Approach and prosthesis at discretion of the operating surgeon. | 17 | Approach and prosthesis at discretion of the operating surgeon. |
| Cadossi 2013  (+ Giannini 2011) | Italy | 1 | 83 | Age >70, walking independently without aids. | 41 | Direct lateral approach. Cemented or uncemented Exeter femoral component with a bipolar femoral head. | 47 | Direct lateral approach. Uncemented Conus stem with a large-diameter femoral head and a polycarbonate-urethane (PCU) acetabular component. |

*In addition to displaced intracapsular hip fractures, which was an inclusion criterion common to all included trials.

| **Table S3: Risk of bias assessments for included studies** | | | | |
| --- | --- | --- | --- | --- |
| **Study** | **Type of bias** | **Domain** | **Assessment** | **Reason** |
| Baker 2006  (+ Avery) | Selection bias  Performance bias  Detection bias  Attrition bias  Reporting bias  Other bias | Random sequence generation  Allocation concealment  Blinding of participants  Blinding of outcome assessment  Incomplete outcome data  Selective reporting  Other | Low  High  High  High  Low  Unclear  Low | Sealed envelopes.  Sealed envelopes without additional precautions.  Not blinded.  Not blinded.  1/41 patients declined follow-up.  No protocol published.  None. |
| Keating 2006 | Selection bias  Performance bias  Detection bias  Attrition bias  Reporting bias  Other bias | Random sequence generation  Allocation concealment  Blinding of participants  Blinding of outcome assessment  Incomplete outcome data  Selective reporting  Other | Low  Low  High  High  Unclear  Unclear  Low | Computer-based telephone randomization.  Computer-based telephone randomization.  Not blinded.  Not blinded.  Not stated.  No protocol published.  None. |
| Blomfeldt 2007  (+ Hedbeck 2011) | Selection bias  Performance bias  Detection bias  Attrition bias  Reporting bias  Other bias | Random sequence generation  Allocation concealment  Blinding of participants  Blinding of outcome assessment  Incomplete outcome data  Selective reporting  Other | Low  High  High  High  Low  Unclear  Low | Sealed envelopes.  Sealed envelopes without additional precautions.  Not blinded.  Not blinded.  2/120 patients lost to follow-up.  No protocol published.  None. |
| Macaulay 2008 | Selection bias  Performance bias  Detection bias  Attrition bias  Reporting bias  Other bias | Random sequence generation  Allocation concealment  Blinding of participants  Blinding of outcome assessment  Incomplete outcome data  Selective reporting  Other | Low  Low  High  High  Unclear  Unclear  Low | Sealed envelopes.  Sealed envelopes with additional precautions.  Not blinded.  Not blinded.  Not stated.  No protocol published.  None. |
| Cadossi 2013  (+ Giannini 2011) | Selection bias  Performance bias  Detection bias  Attrition bias  Reporting bias  Other bias | Random sequence generation  Allocation concealment  Blinding of participants  Blinding of outcome assessment  Incomplete outcome data  Selective reporting  Other | Low  High  High  High  Low  Unclear  Low | Sealed envelopes.  Sealed envelopes without additional precautions.  Not blinded.  Not blinded.  All patients followed up.  No protocol published.  None. |
