# Supplementary material for: Total hip arthroplasty versus hemiarthroplasty for independently mobile older adults with intracapsular hip fractures
Source: BMC Musculoskelet Disord. 2019 May 17;20:226. doi: 10.1186/s12891-019-2590-4 (PMC6525472; doi:10.1186/s12891-019-2590-4)
Supplement: Supplementary file 3 — Table S1. Characteristics of the unmatched population. Figure S1. Histograms showing the distribution of propensity scores before and after matching. Figure S2. Quantile-quantile plots of co-variables between the two groups before and after matching. Data from populations with the same empirical distribution will lie along the 45 degree reference line. Figure S3. Co-variables plotted against propensity scores by treatment status. If the two are identical, this indicates that the groups have the same mean for each value of the propensity score and so are well matched. Figure S4. A jitter plot showing the overall distribution of propensity scores for both matched and unmatched records. (DOCX 689 kb) [file 12891_2019_2590_MOESM3_ESM.docx]

**Additional file 3: Appendix 3**

| **Table S1: Characteristics of the unmatched population** | | | |
| --- | --- | --- | --- |
|  | **Hemiarthroplasty** | **Total hip arthroplasty** | **Total** |
| **Age*** | 83 (78-87) | 73 (68-78) | 79 (73-85) |
| **Sex****  Male  Female | 3,607 (23.1%)  11,991 (76.9%) | 2,178 (21.4%)  7,980 (78.6%) | 5,785 (22.5%)  19,971 (77.5%) |
| **ASA*** | 2 (2-2) | 2 (2-2) | 2 (2-2) |
| **Pre-injury mobility****  Independently mobile  Mobile indoors with one aid | 10,190 (65.3%)  5,408 (34.7%) | 9,264 (91.2%)  894 (8.8%) | 19,454 (75.5%)  6,302 (24.5%) |
| **AMTS*** | 10 (9-10) | 10 (10-10) | 10 (10-10) |
| **Admission source****  Own home  Rehabilitation unit  Residential/nursing home  Acute hospital | 15,298 (98.1%)  19 (0.1%)  173 (1.1%)  108 (0.7%) | 10,089 (99.3%)  2 (0.0%)  24 (0.2%)  43 (0.4%) | 25,387 (98.6%)  21 (0.1%)  197 (0.8%)  151 (0.6%) |

*Median (interquartile range); **number (percentage).

**Figure S1: Histograms showing the distribution of propensity scores before and after matching.**

**
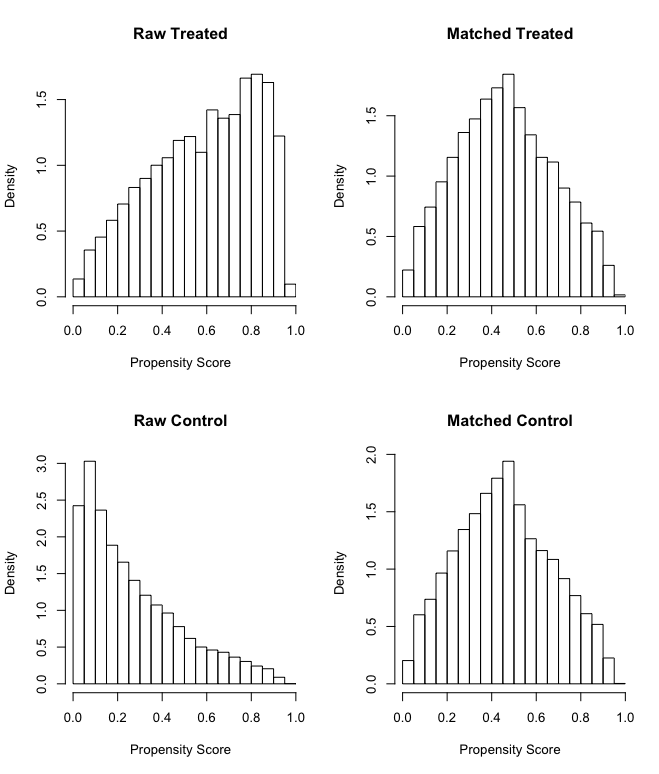
**

**Figure S2: Quantile-quantile plots of co-variables between the two groups before and after matching.** *Data from populations with the same empirical distribution will lie along the 45 degree reference line.*

**
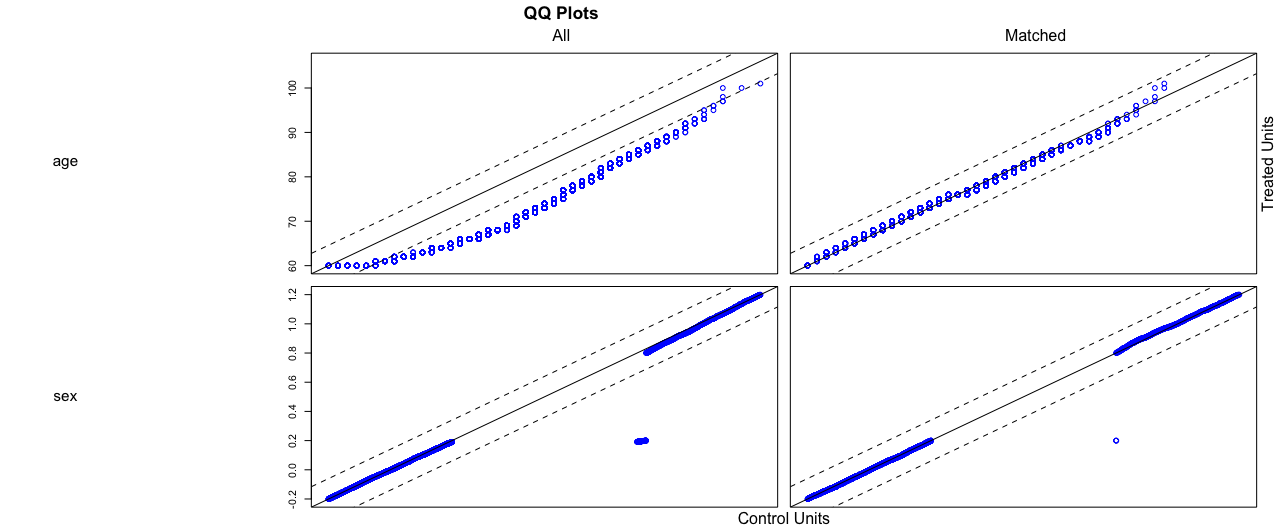
**

**
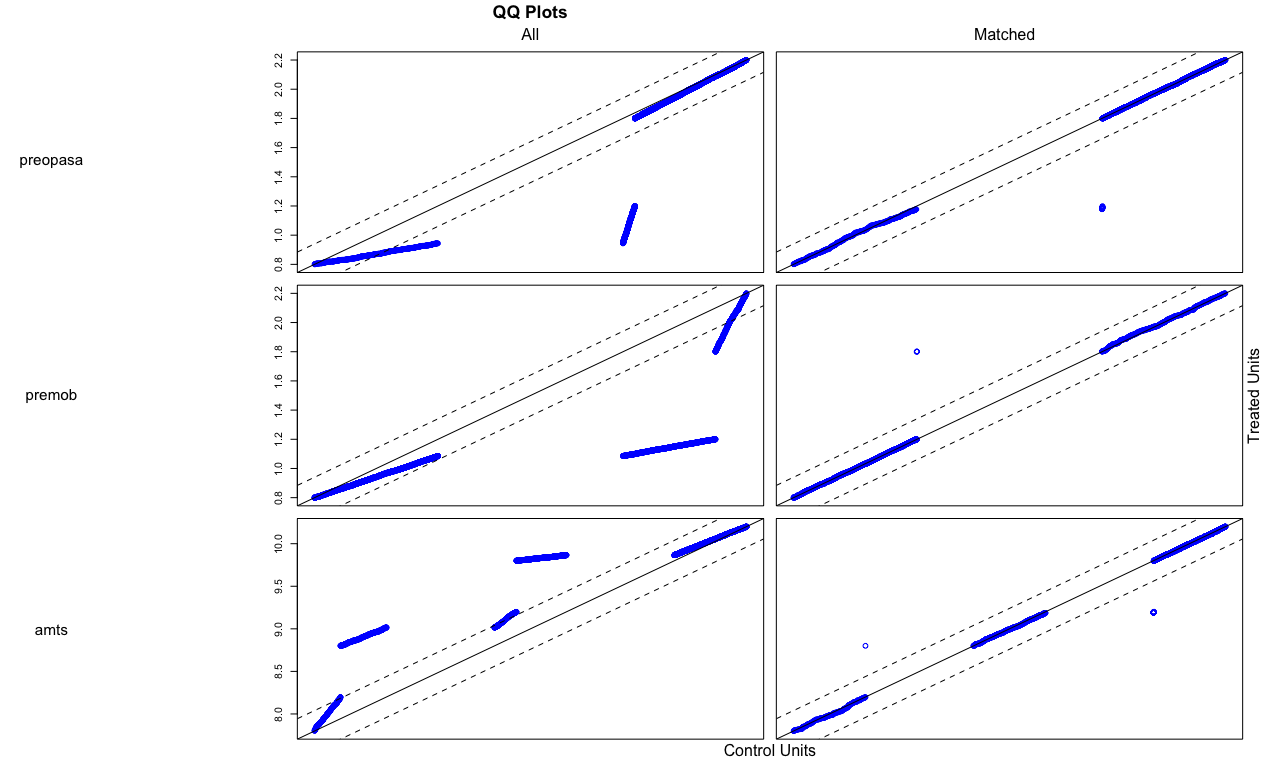
**

**
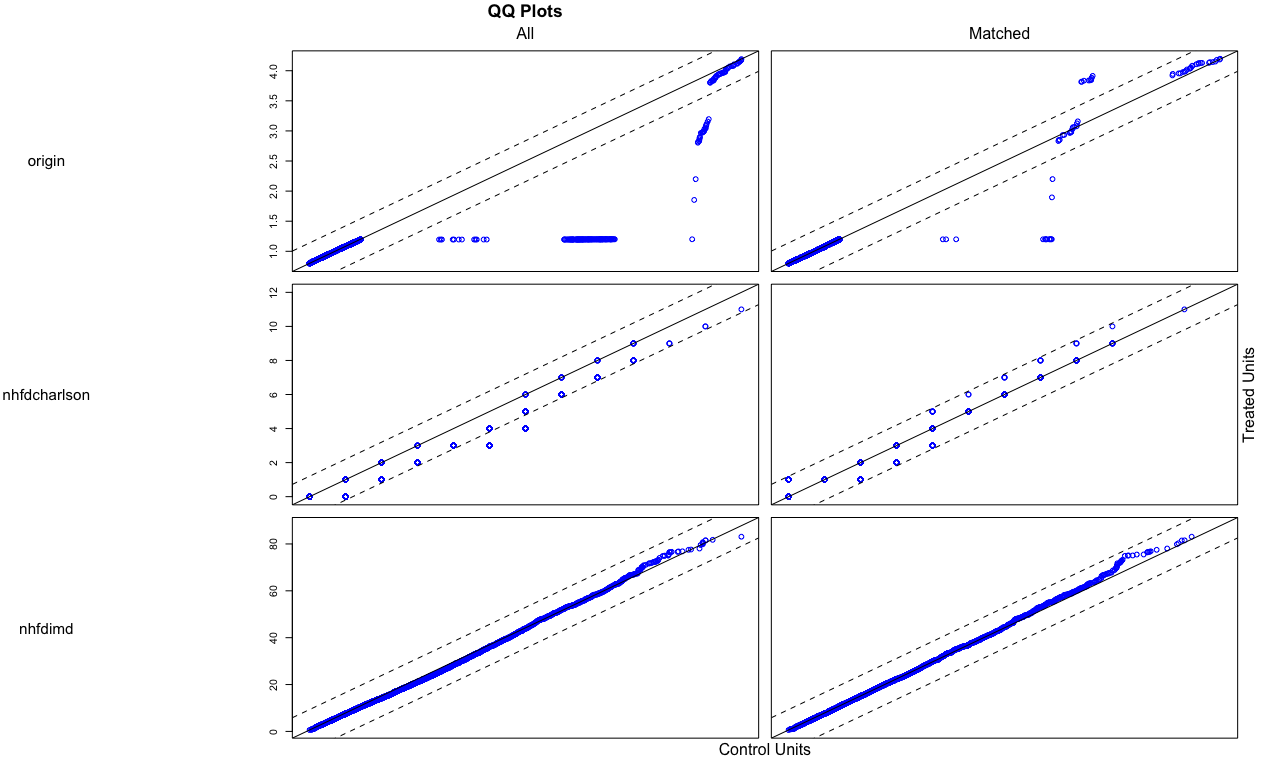
**

**Figure S3: Co-variables plotted against propensity scores by treatment status.** *If the two are identical, this indicates that the groups have the same mean for each value of the propensity score and so are well matched.*

**
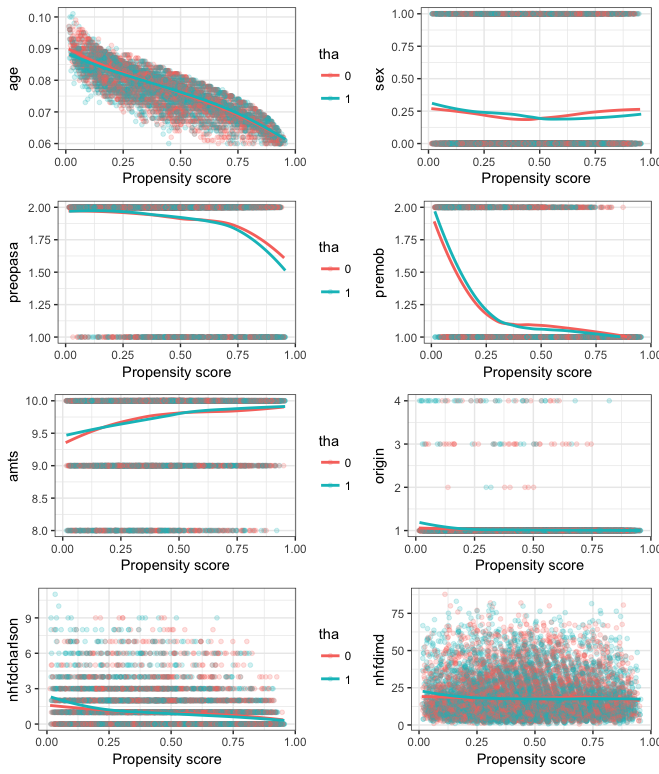
**

**Figure S4: A jitter plot showing the overall distribution of propensity scores for both matched and unmatched records.**

**
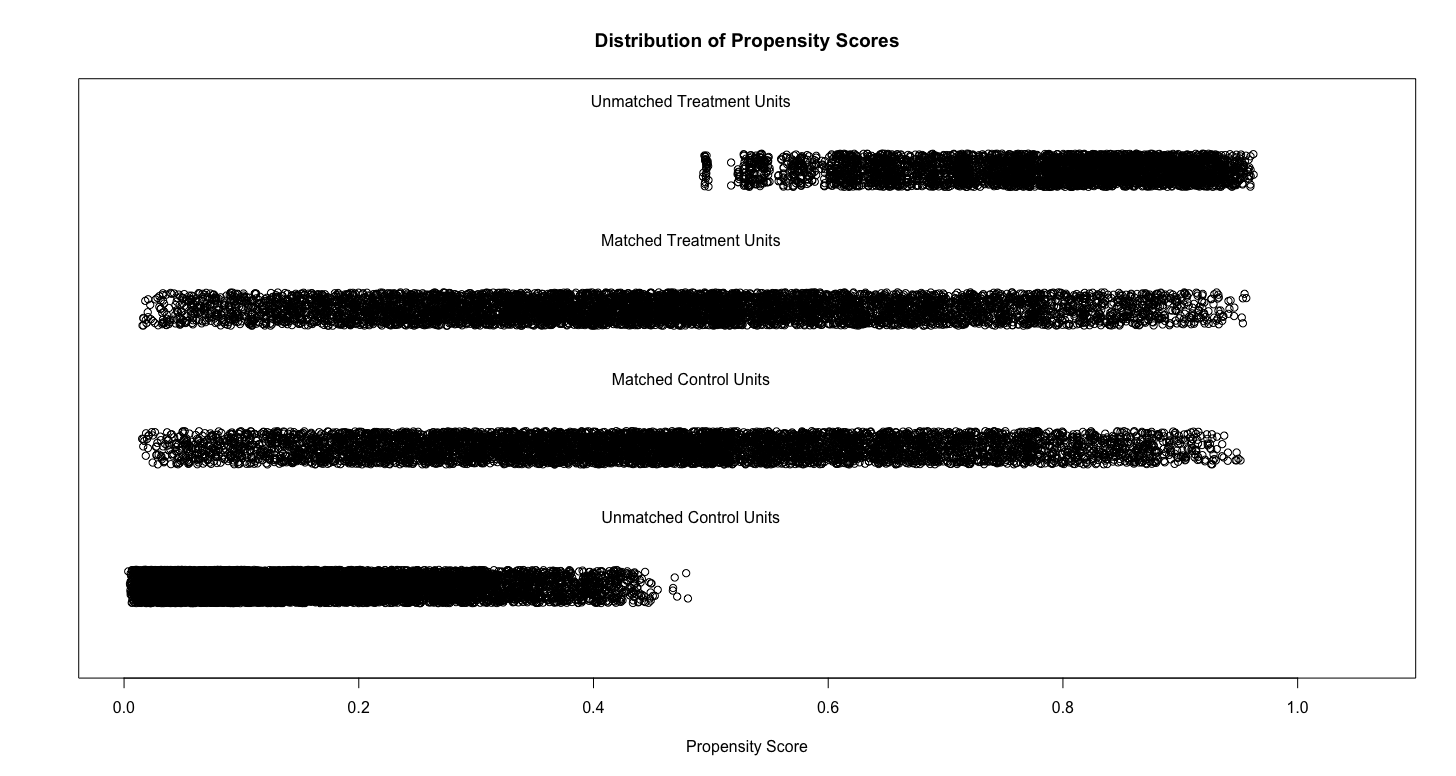
**
